# Intrinsic Cerebro-Cerebellar Functional Connectivity Reveals the Function of Cerebellum VI in Reading-Related Skills

**DOI:** 10.3389/fpsyg.2020.00420

**Published:** 2020-03-20

**Authors:** Chen Ang, Jia Zhang, Mingyuan Chu, Hehui Li, Mengyu Tian, Xiaoxia Feng, Manli Zhang, Li Liu, Xiangzhi Meng, Guosheng Ding

**Affiliations:** ^1^State Key Laboratory of Cognitive Neuroscience and Learning and IDG/McGovern Institute for Brain Research, Beijing Normal University, Beijing, China; ^2^School of Psychology, University of Aberdeen, Aberdeen, United Kingdom; ^3^School of Psychological and Cognitive Sciences, Beijing Key Laboratory of Behavior and Mental Health, Peking University, Beijing, China; ^4^PekingU-PolyU Center for Child Development and Learning, Peking University, Beijing, China

**Keywords:** cerebellum VI, resting state functional connectivity, fMRI, phonological awareness, rapid automatized naming

## Abstract

The engagement of the cerebellum VI in reading was reported in both typically developing and dyslexic readers. However, it is still not clear how the cerebellum VI contributes to reading. Here we have examined the correlation of intrinsic cerebro-cerebellar functional connectivity with two critical reading-related skills—phonological awareness (PA) and rapid automatized naming (RAN)—with fMRI technology. Specifically, we tested the hypothesis that the cerebellum may contribute to reading either by phonological skills or by automatizing skills. We chose the left and right cerebellum VI as ROIs, and we calculated the intrinsic cerebro-cerebellar functional connectivity during a resting state. We further explored whether and how cerebro-cerebellar resting state functional connectivity (RSFC) is associated with individuals’ reading-related skills including PA and RAN. The results showed that the functional connectivity between the left supramarginal gyrus and bilateral cerebellum VI was related to RAN, and the connectivity between the left insula and right cerebellum VI was related to PA. However, the effect of PA did not survive after the RAN was regressed out. Control analyses further confirmed that it was the intrinsic cerebro-cerebellar functional connectivity rather than the local cerebellar functionality that associated with phonological awareness ability and rapid automatized naming ability. For the first time, the relationship between cerebro-cerebellar resting state functional connectivity and specific reading-related skills has been explored, and this has deepened our understanding of the way the cerebellum VI is involved in reading.

## Introduction

Increasing evidence has shown that the cerebellum is engaged in high-level cognitive processing, particularly, in reading (Baillieux et al., 2008; Stoodley and Stein, 2011, 2013; Argyropoulos, 2016; Sokolov et al., 2017). For example, numerous studies have reported that the cerebellum was involved in a variety of reading tasks, including phonological processing, visual letter recognition, as well as semantic processing (McDermott et al., 2003; Turkeltaub et al., 2003; Booth et al., 2007; King et al., 2019). Particularly, the bilateral cerebellum VI, a middle part of the cerebellum, plays a more essential role than other parts in reading. A voxel-based morphometry study found a significant negative correlation between the gray matter volume of the left cerebellum VI (lobule VI/Crus I) and reading accuracy in normal readers (Jednorog et al., 2015). A meta-analysis also found that the right cerebellum VI was consistently activated during reading-related tasks (Martin et al., 2015). Although these findings suggest a relationship between the bilateral cerebellum VI and reading, it remains unclear how the cerebellum VI contributes to reading.

The involvement of the cerebellum in high-level cognitive processing could be attributed to the structural and functional cerebro-cerebellar connection (Sokolov et al., 2017). As a support, Booth et al. (2007) observed that the cerebellum VI functionally connected with cerebral regions during reading, including the left fusiform gyrus, the left inferior frontal gyrus, and the left lateral temporal cortex. Feng et al. (2017) further found that abnormality of cerebro-cerebellar connections was associated with reading impairment, suggesting that variation of cerebro-cerebellar functional connectivity is associated with differences in reading ability.

A previous study indicated that an intrinsic network organization underlying cognitive processes can be reflected by resting state functional connectivity (RSFC) (Lohmann et al., 2009). For example, RSFC is associated with individual differences in several cognitive domains, including executive control, episodic memory, and learning (Wang et al., 2010; Xu et al., 2014; Chai et al., 2016). Compared to functional connectivity during certain cognitive task, RSFC indicates intrinsic, task-independent features of brain function (Biswal et al., 1995; Fox et al., 2005). Importantly, task-induced BOLD activity could be predicted by RSFC (Mennes et al., 2010; Shah et al., 2016), and the reading network can also be investigated during the resting state (Koyama et al., 2010). Koyama et al. (2011) found that reading competence positively related to RSFC between the left precentral gyrus and other motor regions as well as between the pars opercularis of the left inferior frontal gyrus and the posterior part of the left superior temporal gyrus, suggesting that reading can be facilitated by stronger connectivity among motor regions and between language regions. Besides, it was proposed that RSFC was associated with Chinese reading abilities (Wang et al., 2012; Zhang et al., 2014; Qian et al., 2016). A recent study further observed that the strength of RSFC between the left thalamus and the right cerebellum, which are thought to be associated with attention, is positively correlated with phonological fluency (Miro-Padilla et al., 2017). However, up until now, no study has directly explored whether RSFC between the cerebellum and cerebrum is associated with reading.

As to the mechanism of how the cerebellum is engaged in reading, the cerebellar deficit hypothesis proposed that articulatory processing and automatizing processing were two key components that the cerebellum possibly contributes toward, the deficit of which can lead to subsequent problems when learning how to read (Nicolson et al., 2001). Moreover, lack of articulatory fluency will finally lead to difficulties in phonological awareness (Nicolson et al., 2001). Evidence to support this hypothesis comes from two aspects. Firstly, cerebellum was indeed engaged in phonological (Raschle et al., 2012; Meng et al., 2016) and automatizing processing (Norton et al., 2014; Cummine et al., 2015). Secondly, phonological skill and automatizing skill are closely related to reading. For example, phonological awareness (PA) and rapid automatized naming (RAN, a way to estimate automatizing skill) have been confirmed as significant predictors of reading performance across languages (Parrila et al., 2004; Ziegler et al., 2010; Yeung et al., 2011). A meta-analysis further revealed that PA and RAN were correlated to reading accuracy and reading fluency, respectively (Song et al., 2015). These two skills have frequently been used to evaluate reading abilities (Qian et al., 2015, 2016) and distinguish between dyslexic and normal readers (Norton et al., 2014).

The cerebellar deficit hypothesis also indicated that reading impairment can be caused by cerebellar deficits showing up before reading acquisition, and the cerebellum plays a vital role in the initial stage of reading (Nicolson et al., 2001). A meta-analysis has reported both common and divergent reading-related activation in children and adults (Martin et al., 2015), and a previous study also proposed that reading competence was related to different RSFC patterns in children and adults (Koyama et al., 2011). Koyama et al. (2011) demonstrated that better reading performance was associated with stronger functional coupling between the fusiform gyrus and phonology-related regions/default mode network in adults but not in children. The research to date has tended to focus on RSFC-reading relationships in adults (Wang et al., 2012; Zhang et al., 2014; Qian et al., 2016). However, far too little attention has been paid to the relationship between RSFC and reading in children (Alcauter et al., 2017). Given reading skills are not yet fully matured during childhood, studies based on children can investigate the way the cerebellum affects reading in the developmental stages.

The current study has aimed to examine whether and how the intrinsic cerebro-cerebellar functional connectivity (RSFC) associates with the two important reading-related skills—phonological and automatizing skills—in children. For this purpose, we have analyzed the correlation between intrinsic cerebro-cerebellar functional connectivity with PA and RAN, both of which are important predictors of reading performance. Given the convergent evidence, which showed that the cerebellum VI was consistently involved in reading, we chose bilateral cerebellum VI as regions of interest (ROIs). We hypothesized that reading-related cognitive processes were supported by the cooperation of the cerebellum and cerebrum. Specifically, both PA and RAN scores were associated with the intrinsic functional connectivity between the cerebellum VI and cerebral areas.

## Materials and Methods

### Participants

Fifty-seven typically developing children without reading disorders [31 males and 26 females, mean age = 10.19 years, standard deviation (SD) = 0.96] took part in the experiment. All participants were native speakers of Mandarin and were recruited and screened from several primary schools in Beijing from grades three to six. They were all right-handed and had normal IQs (Raven Percentiles ≥50, Raven’s Standard Progressive Matrices: Raven, 1998) with normal or corrected-to-normal vision. In addition, children with attention deficit hyperactivity disorder (ADHD, Feng et al., 2017), neurological disease, or psychiatric disorders were excluded. All the 57 participants meet the criteria of head motion not exceeding 3 mm or 3°. This study was approved by the Institutional Reviews Board of the State Key Laboratory of Cognitive Neuroscience and Learning at Beijing Normal University, and written consent was obtained from the children and their parents.

### Behavior Measures

Raven’s Standard Progressive Matrices was applied to test the children’s IQ. A Chinese phonological awareness test (Shu et al., 2006, 2008) and a rapid automatized naming test (Feng et al., 2017) were then used to evaluate the children’s phonological and automatizing skills. Details of the two tests are described below.

#### Chinese Phonological Awareness (PA) Test

The Chinese phonological awareness test consists of four subtests: phoneme deletion, tone detection, onset detection, and rime detection. In the phoneme deletion test, the participants were asked to pronounce a given word after deleting a phoneme, for example, “shua3” after removing “a” should be pronounced as “shu3.” In the tone/onset/rime deletion test, the participants were asked to find the one word, out of four words, that differed (by the tone, onset, or rime level). For example, when it comes to the tone level, such as [ba4, san4, bei4, bo1], the different one is bo1; for the onset detection, [ba3, san1, bei4, bo1], the correct answer is san1; and for the rime detection, [ban3, san1, ban4, bo1], bo1 was the different one. The number of correct answers was recorded as a raw score. Raw scores were firstly converted into *Z* scores in each grade and then into standard scores with a mean of 100 and SD of 15. Averaged *Z* scores of the four tests indicated the phonological processing ability. The higher the score, the better the phonological awareness.

#### The Rapid Automatized Naming (RAN) Test

The rapid automatized naming test was used to measure automatization ability (Raberger and Wimmer, 2003). When performing this task, participants were required to read out visually-presented Arabic numbers as quickly and accurately as possible. The participants did the test twice, and the total time to read all digits was recorded each time. The average time was calculated and translated into *Z* scores in each grade. The *Z* scores were then multiplied by −1, and they were then converted into standard scores with a mean of 100 and SD of 15; higher scores represented better performances.

### Imaging Procedure and Acquisition

Resting-state images were acquired with a 3T Siemens scanner at Beijing Normal University. Before the formal experiment, the participants participated in 20 min of training in a mock scanner in order to familiarize themselves with the environment and the requirements. The resting-state MRI scanning session lasted for 8 min. The participants were informed to close their eyes, keep their head and body still, and to think of nothing to avoid inner language disturbances. A T2-weighted gradient-echo EPI sequence was used to acquire functional images, and the acquisition parameters were TR = 2400 ms, TE = 30 ms, flip angle = 81°, FOV = 192 × 192 mm, slice number = 40 slices, slice thickness = 3 mm, and voxel size = 3 × 3 × 3 mm. We used Sequential scanning. For better image registration, we also collected T1-weighted images, TR = 2300 ms, TE = 4.18 ms, flip angle = 9°, FOV = 256 × 256 mm, slice number = 176 slices, slice thickness = 1 mm, and voxel size = 1 × 1 × 1 mm.

### Data Analysis

#### Preprocessing

Functional MRI scans were preprocessed with DPABI software^1^ (Yan et al., 2016). The resting state functional image preprocessing included several steps: (1) the first 10 time points were deleted; (2) the slice-timing was corrected with the middle slice as the reference slice and realignment; (3) all functional images were co-registered to the corresponding anatomical image; (4) functional images were normalized to Montreal Neurological Institute (MNI) space; (5) the spatial was smoothed with 4 mm FWHM Gaussian kernel; (6) linear trends were removed, and band-pass temporal filtering (0.01–0.08 Hz) was applied. In addition, we regressed out six motion parameters, the white matter signal, and the cerebrospinal fluid signal to reduce motion and physiological signal interference.

#### Cerebellum ROI

**FIGURE 1:**
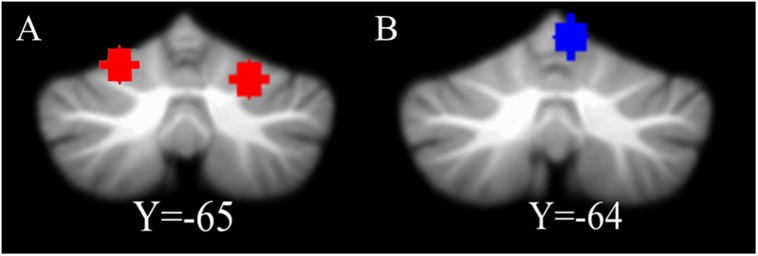
The location of ROIs. **(A)** The coordinate of the left cerebellum VI is [–22, –68, –20] and the right cerebellum VI is [24, –66, –24]. **(B)** The location of control ROI. The right cerebellum V [6, –64, –10], a motion-related region, is used for a control analysis.

Coordinates of the bilateral cerebellum VI were chosen based on a previous meta-analysis (Keren-Happuch et al., 2014). The sphere of the left cerebellum VI was centered at MNI coordinate (−22, −68, −20) and the right cerebellum VI was centered at MNI coordinate (24, −66, −24, Figure 1A). For each ROI, a sphere was created with a radius of 6 mm.

In addition, we chose the cerebellum V (MNI coordinate [6, −64, −10], Figure 1B; Stoodley and Schmahmann, 2009), related to motor function, as another ROI to use to perform a control analysis (for detailed information, see section “Control analyses”). For the display, the cerebellum ROIs were overlaid onto the suit template^2^ with the software MRIcron^3^.

#### Correlation of Cerebro-Cerebellar RSFC and Reading-Related Skills

We firstly extracted and averaged the BOLD signal of all voxels within each ROI (i.e., left or right cerebellum VI) used as the seed region for functional connectivity analysis. The time-series correlation of average BOLD signal between each seed region and each voxel in the bilateral cerebrum was calculated with SPM12^4^. The correlation coefficients were transformed into Fisher’s *Z* scores as an index for cerebro-cerebellar RSFC. Finally, we calculated the correlation between *Z* scores and the phonological or rapid naming test performances in each seed region.

All the correlation maps were corrected for multiple comparisons by Gaussian random field (GRF) correction (voxel-level *p* < 0.001 and cluster-level *p* < 0.05, two-tailed) with DPABI software (see text Footnote 1; Yan et al., 2016). GRF corrections were widely used to control the family-wise error rate (FWER) of testing multiple hypothesis in neuroimaging. Chen et al. (2018) confirmed that the FWER can reach the nominal 5% level if the threshold was set voxel-wise *p* < 0.0005 and cluster-wise *p* < 0.025 to perform two one-tailed tests, which is equivalent to the threshold in our study (voxel-level *p* < 0.001 and cluster-level *p* < 0.05, two-tailed). This multiple comparison correction method was recommended when considering the FWER (Chen et al., 2018). In addition, the peak voxel in each cluster was reported based on the Automated Anatomical Labeling (AAL) template (Tzourio-Mazoyer et al., 2002). All threshold brain images were overlaid onto the Brainmesh_ICBM152_smoothed.nv surf template with the software BrainNet Viewer (Xia et al., 2013)^5^ for display.

In our study, we were primarily concerned with how the cerebro-cerebellar RSFC associates with phonological skills and automatizing skills, as indicated by the measures of PA and RAN. We then first calculated the correlation between the cerebro-cerebellar RSFC with each skill separately. Given that there is potential correlation between Raven’s IQ, PA, and RAN, we additionally performed a partial correlation analysis. For details, when we calculated the correlation between cerebro-cerebellar RSFC and RAN, either the Raven’s IQ, the scores of PA, or both were used as the covariate(s). When we calculated the correlation between cerebro-cerebellar RSFC and PA, the Raven’s IQ, the scores of RAN, or both were used as the covariate(s).

#### Control Analyses

We further performed two control analyses to exclude potential confounds or other possibilities. Firstly, given the RSFC might be confounded by the local functionalities of the cerebellum, we calculated the amplitude of low frequency fluctuation (ALFF), a representative index of local brain functionalities in the resting state fMRI (Zang et al., 2007), and explored whether the ALFF of bilateral cerebellum VI correlated to the two reading-related skills. The amplitude of low frequency fluctuation (ALFF) measures the spontaneous activity of the specific brain area in order to reflect the local functionality property. We followed the calculation procedure used in previous studies (Deluca et al., 2014). Fast Fourier transform (FFT) was used to transform the time series to frequency domain. After calculating the power spectrum, it was square rooted, and the square root across 0.01–0.08 Hz was then averaged at each voxel, and this was taken as the ALFF. The ALFF of each voxel was divided by the mean ALFF value for standardization. At last, the averaged ALFF of each ROI was extracted for further correlation analysis. The significance of correlation was estimated by Bonferroni correction for multiple comparisons.

Secondly, we tested whether the cerebellum V, a region related to motor function, also showed correlation between intrinsic cerebro-cerebellar RSFC and PA/RAN. During this analysis, we extracted the averaged BOLD signal of cerebral regions whose RSFC with the bilateral cerebellum VI correlated with PA or RAN. Then we calculated the RSFC between the cerebellum V and these cerebral regions to explore whether this intrinsic RSFC was associated with the two reading-related skills. This analysis aimed to answer whether the association between intrinsic cerebro-cerebellar RSFC and reading-related skills is just a feature of the cerebellum VI or could be generalized to other cerebellum regions, such as the cerebellum V. In this analysis, we replaced the cerebellum VI with cerebellum V. The significance of correlation was also estimated by Bonferroni correction for multiple comparisons.

## Results

### Behavior Results

Table 1 shows the demographic information and the results of reading-related tests. The scores of both the phonological awareness test and the rapid automatized naming test followed the normal distribution. The correlation between the scores of PA and RAN was significant (*r* = 0.41, *p* = 0.002) with the Bonferroni correction. The correlation was marginally significant between Raven’s IQ and PA (*r* = 0.232, *p* = 0.082) and not significant between Raven’s IQ and RAN (*r* = 0.220, *p* = 0.101).

**TABLE 1:** Characteristics of participants.

	Participants (*n* = 57)	
	Mean (SD)	Score range
Age	10.19 (0.95)	8–12
Gender (male/female)	31:26	
Grade (3/4/5/6)	9/25/17/6	
Handedness	All right-handed	
Raven’s IQ^a^	78.68 (12.96)	50–95
**Chinese reading-related tests^b^**		
Phonological awareness test (PA)	103.23 (8.89)	81.10–118.57
Rapid automatized naming test(RAN)	98.46 (8.33)	72.67–117.10

*SD = standard deviation. ^a^Percentiles.^b^Standard scores.*

### Correlation of Cerebro-Cerebellar RSFC and Reading-Related Skills

**FIGURE 2:**
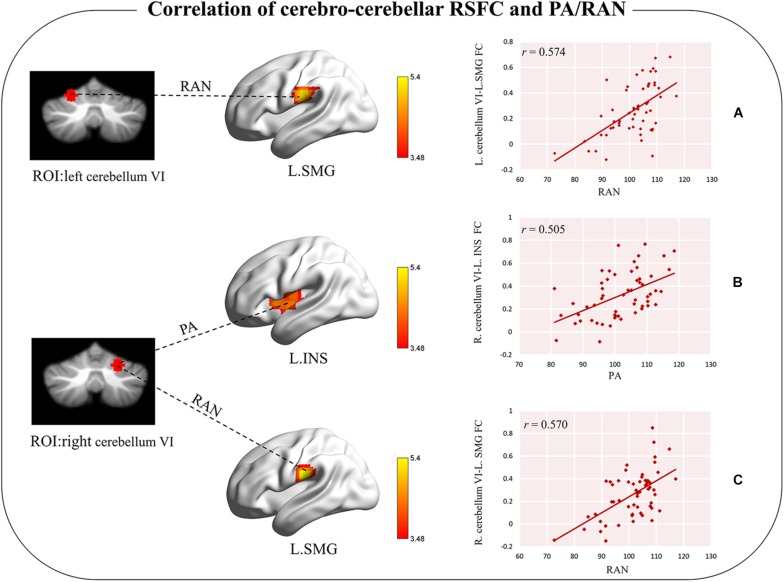
Significant correlation between cerebro-cerebellar functional connectivity and PA/RAN. **(A)** Functional connectivity between left cerebellum VI and left SMG was positively correlated with RAN. **(B)** Functional connectivity between right cerebellum VI and left INS was positively correlated with PA. **(C)** Functional connectivity between right cerebellum VI and left SMG was positively correlated with RAN under a loose threshold. L.SMG, the left supramarginal gyrus; L.INS, the left insula; RAN, rapid automatized naming; PA, phonological awareness.

When the left cerebellum VI was used as a seed region, the RSFC of this ROI and the left supramarginal gyrus (extended to the postcentral gyrus) was significantly correlated with RAN scores (cluster-level GRF corrected *p* < 0.05, voxel-level *p* < 0.001; cluster size = 91, Table 2 and Figure 2A), which revealed that better RAN performance related to stronger functional connectivity. No significant correlation was observed between the PA scores and the RSFC (cluster-level GRF corrected *p* < 0.05, voxel-level *p* < 0.001).

**TABLE 2:** The correlation between the cerebro-cerebellar resting-state functional connectivity and PA/RAN.

Seed regions	Reading-related tests	Cerebral cortex	Lateral	MNI coordinates			*T* value	Voxels
				x	y	Z		
Left cerebellum VI	RAN	Supramarginal gyrus	Left	−63	−21	27	5.13	91
Left cerebellum VI	PA	/						
Right cerebellum VI	PA	Insula	Left	−57	−3	3	4.69	80
Right cerebellum VI	RAN	*Supramarginal gyrus	Left	−57	−18	24	5.40	62

T-value, and MNI coordinate are for the peak voxel in each cluster only. Results were reported at a threshold of an individual voxel-level p < 0.001, cluster-level p < 0.05, corrected by GRF. *a loose threshold of an individual voxel p < 0.001 uncorrected. RAN = rapid automatized naming, PA = phonological awareness.

When the right cerebellum VI was used as a seed, the RSFC between the ROI and the left insula (extended to the superior temporal gyrus) was found to be correlated with PA (cluster-level GRF corrected *p* < 0.05, voxel-level *p* < 0.001, cluster size = 80, Table 2 and Figure 2B), suggesting that better PA performance also related to stronger functional connectivity. No significant correlation between RAN scores and RSFC was observed at this stringent threshold. However, we did observe a tendency for the RSFC between the ROI and the left supramarginal gyrus (extended to the postcentral gyrus) to correlate with RAN, which could survive with a loose threshold (voxel-level *p* < 0.001, uncorrected, cluster size = 62, Table 2 and Figure 2C).

In order to exclude the potential effect of IQ on the results, we re-did the above brain and behavior correlation analyses with Raven’s IQ as the covariate. When Raven’s IQ was regressed out, the performance on RAN still correlated with the RSFC between the left cerebellum VI and left postcentral gyrus (extended to the supramarginal gyrus) and between the right cerebellum VI and left supramarginal gyrus (extended to the postcentral gyrus), using the corrected threshold as in the above analysis. But we can only observe the correlation of PA and RSFC between the right cerebellum VI and the left insular at a looser threshold (voxel-level *p* < 0.001, uncorrected, Supplementary Table S1 and Supplementary Figure S1).

Furthermore, when PA was regressed out, the performance on RAN was correlated with the RSFC between the left cerebellum VI and left supramarginal gyrus (extended to the postcentral gyrus) and between the right cerebellum VI and left supramarginal gyrus (extended to the postcentral gyrus) at the threshold of voxel-level *p* < 0.001, uncorrected. When RAN was used as a covariate, the correlation between PA and RSFC of the right cerebellum VI with the left insula did not survive, even with an uncorrected threshold (voxel-level threshold *p* < 0.001, uncorrected, Supplementary Table S2 and Supplementary Figure S2).

Finally, when PA and Raven’s IQ were regressed out, the correlation of RAN and the RSFC between the left cerebellum VI and left supramarginal gyrus (extended to the postcentral gyrus) and between the right cerebellum VI and left supramarginal gyrus (extended to the postcentral gyrus) could be observed with an uncorrected threshold (voxel-level *p* < 0.001, uncorrected). When RAN and Raven’s IQ were regressed out, the correlation between PA and RSFC did not survive even with an uncorrected threshold (voxel-level threshold *p* < 0.001, uncorrected; Supplementary Table S3 and Supplementary Figure S3).

Based on the above results, it is likely that the RSFC of the cerebellum VI does not directly contribute to PA. Instead, RSFC might be affected by PA via RAN. We then further conducted a mediation analysis to test this possibility. However, we did not observe a significant mediation effect of RAN for the cerebellum VI functional connectivity. Detailed information is displayed in the Supplementary Figure S4.

### Control Analyses

**FIGURE 3:**
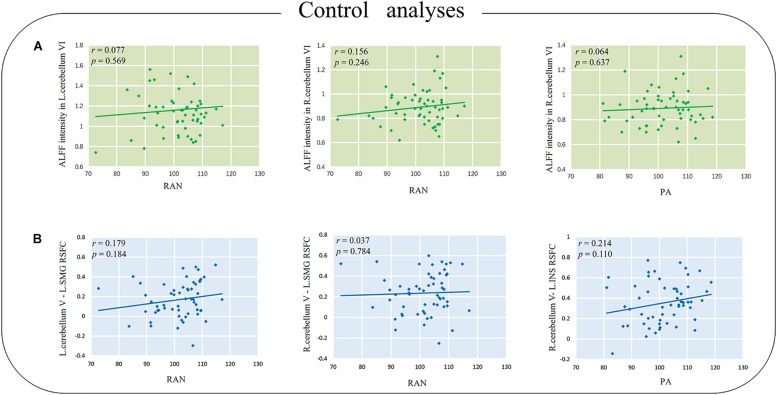
**(A)** The correlations between ALFF intensity in the cerebellum and reading-related skills. **(B)** The correlations between cerebellum V–cerebrum RSFC and the reading-related skills. L.SMG, the left supramarginal gyrus; L.INS, the left insula; RAN, rapid automatized naming; PA, phonological awareness; RSFC, resting state functional connectivity. The *p* value in the picture is the uncorrected *p* value.

For ALFF, no significant correlation was found between either the left or right cerebellum VI and reading-related skills in our study (*p*s > 0.2). Detailed correlation coefficients (*r*) and *p* values are presented in Figure 3A.

Additionally, the RSFCs of cerebellum V (MNI coordinate [6, −64, −10]) and cerebral regions, including the supramarginal gyrus (MNI coordinate [−63, −21, 27] and [−57, −18, 24]) and the left insular (MNI coordinate [−57, −3, 3]), were not significantly correlated with either reading-related skills (*p*s > 0.1, Figure 3B).

## Discussion

In the current study, we have aimed to investigate the way in which the cerebellum VI contributes to reading. More specifically, we tested whether the cerebro-cerebellar functional connectivity during a resting state (RSFC) was associated with two essential reading-related skills: phonological awareness and rapid automatized naming. To this end, we chose the left and right cerebellum VI as ROIs based on a meta-analysis, and we calculated the correlation of cerebro-cerebellar RSFC and phonological awareness or rapid automatized naming. Our study showed that the connectivity between the left supramarginal gyrus and bilateral cerebellum VI was related to rapid automatized naming, and the connectivity between the left insula and right cerebellum VI was related to phonological awareness. But the latter effect did not survive after the rapid automatized naming was regressed out. The control analyses further showed there is no significant correlation between the local functionality of the cerebellum VI (indicated with ALFF) and both reading-related skills, confirming the association between the intrinsic cerebro-cerebellar functional connectivity and reading-related skills cannot be interpreted by the local functionality of the cerebellum VI. The control analyses also showed that reading-related skills were not correlated with the RSFC between the cerebellum V and cerebral regions, illustrating how the observed intrinsic cerebro-cerebellar RSFC and reading-related skills were just features of the cerebellum VI. For the first time, we tested the hypothesis that the cerebellum contributes to reading, either by phonological skills or by automatizing skills through RSFC, which provided new findings as to how the cerebellum VI contributes to reading.

It is worth noting that reading is a complex process, including not only the more basic levels of linguistic functions (phonological processing and lexical access) involved in reading but also the levels of conceptualization and situation model building (Zacks and Ferstl, 2016). Our study only focused on the former level. Previous studies frequently found the cerebellum VI is involved in reading (Booth et al., 2007; Stoodley and Stein, 2013; Martin et al., 2015; Hancock et al., 2017). More specifically, the association between the cerebellum and rapid automatized naming has been reported in previous studies (Norton et al., 2014), and the abnormity in the cerebellar-frontal circuit was found to be related to rapid automatized naming (Eckert et al., 2003). Rapid automatized naming was also associated with Chinese reading accuracy and fluency (Liao et al., 2007, 2015). Moreover, Cummine et al. (2015) has shown that RAN and reading rely on similar brain regions. Here we have illustrated that the cerebellum VI is involvd in reading through cerebro-cerebellar connections. Specifically, we found a positive relationship between cerebro-cerebellar RSFC and individuals’ performance in RAN. Our findings have provided the first evidence that the intrinsic connectivity during a resting state could be a predictive index for rapid automatized naming skills.

The results of a partial correlation analysis were similar to that of the main analysis when Raven’s IQ was used as a covariate, suggesting such correlations were not affected by Raven’s IQ. We found that the correlation between the left cerebellum VI-left supramarginal gyrus RSFC and PA can survive when PA was regressed out at the threshold voxel-level (*p* < 0.001 uncorrected). Importantly, when PA was regressed out, we also found RAN correlated with RSFC between the right cerebellum VI and left supramarginal gyrus (voxel-level *p* < 0.001, uncorrected), although such an effect was unable to survive after a GRF multiple comparison correction in the main analysis. These findings suggest that there is a relationship between the bilateral cerebellum VI and the RAN. Importantly, when both the RAN and Raven’s IQ were regressed out, we did not observe PA correlated with RSFC between the right cerebellum VI and left insula, even at an uncorrected threshold (voxel-level *p* < 0.001). As proposed by the double-deficit hypothesis, PA and RAN would play a relatively independent role in reading (Wolf and Bowers, 1999). Our findings did not support that the cerebellum VI was equally associated with PA and RAN. After all, the correlation between RSFC of the cerebellum and PA would not survive if RAN was used as a covariate, and we also did not observe if the RSFC of the cerebellum VI can be affected by PA via RAN. However, the possibility cannot be excluded that there might be some common components between these two abilities, which are associated with both articulation and automatization.

The examination of RAN and PA provide insights into the understanding of the cerebellar deficit hypothesis of dyslexia, which has proposed that cerebellar deficit could cause both phonological and automatization deficits at the cognitive level (Nicolson et al., 2001). Functional imaging studies have investigated the double-deficit hypothesis of developmental dyslexia by using the phonological awareness and rapid automatized naming (Norton et al., 2014), which found that children with only phonological awareness deficits showed less activation in the left inferior frontal and inferior parietal regions compared to typically developing readers, and children with only a rapid naming deficit showed less activation in the cerebellum compared to typically developing readers. The current study further showed that the RAN–reading relationship may manifest through the RSFC between the cerebellum and cerebral regions, which is consistent with the established role of the speedy processing of the cerebellum. Actually, it has been frequently proposed that the relationship between RAN and reading should be observed (Eckert et al., 2003; Turkeltaub et al., 2003; He et al., 2013; Norton et al., 2014).

Notably, although PA and RAN are predictors of reading abilities, they are also associated with other cognitive functions, such as attention, working memory, and mathematics (Welsh et al., 2010; Pham et al., 2011; Koponen et al., 2013; Yang et al., 2014). Yang et al. (2014) observed the predictive relation between PA and executive attention in Chinese-English bilingual children, thus suggesting the relationship between PA and executive function. Moreover, RAN was considered to mediate the relationship between attention and reading fluency (Pham et al., 2011), suggesting that the attention ability may be associated with RAN. Other researchers have also proposed that the relationship between RAN and reading is due to executive functions, such as working memory and inhibition (Amtmann et al., 2006). Accordingly, it is unclear whether our correlated functional connectivity via PA/RAN could be specifically dedicated to reading alone.

In addition, we obtained the findings by studying Chinese children learning to read Mandarin. An interesting issue is whether the correlation between the cerebro-cerebellar RSFC and RAN could be generalized to alphabetic language. We have speculated that the answer is yes. RSFC exhibited significant positive correlations with reading abilities for Chinese children (Wang et al., 2012; Zhang et al., 2014; Qian et al., 2016). As for alphabetic language, Koyama et al. (2011) also observed that reading competence in children correlated positively with RSFC between the left precentral gyrus and other motor regions as well as between Broca’s and Wernicke’s areas. Even though these studies did not directly examine the relationship between the RSFC of the cerebellum and reading competence, it has been reported that the cerebellum also played an essential role in phonological processing for alphabetic language (Booth et al., 2007; Stoodley and Stein, 2013).

Finally, our findings have provided new evidence for the functional segregation of the cerebellum. Recent studies have shown that the anterior part of the cerebellum is responsible for movement and other low-level processing, while the posterior part of the cerebellum is mainly responsible for high-level cognitive functions, including language, memory, emotion, and so on (Stoodley and Schmahmann, 2009), which suggests that there is a functional segregation of cerebellum. Here we found that the cerebro-cerebellar functional connectivity of the cerebellum VI but not cerebellum V correlated with certain reading-related skill(s), thus confirming the functional differentiation between the cerebellum VI and V. Future studies may be required to comprehensively examine how cerebellum is functionally segregated, and how the different subregions cooperate with cerebrum in reading or other high-level cognitive processing through cerebro-cerebellar functional connectivity.

There are some limitations in our study. Firstly, we performed resting-state functional connectivity rather than task-based, which may not be able to reliably generalize the findings based on RSFC to encompass task-related neural activity; we should be cautious when assessing the relationship between RSFC and reading-related skills (Brock, 2013). Future studies are required to examine the association between task-related cerebro-cerebellar functional connectivity and these reading-related skills. Secondly, a recent meta-analysis has shown that the reliability of RSFC is rather mediocre (Noble et al., 2019), and our results need to be further validated by carrying out more research in the future. In addition, we only looked at RAN and PA without other reading-relevant cognitive factors, and these two tests were associated with other cognitive functions besides reading. The association of the connectivity between brain regions and the cognitive task has to be interpreted cautiously, as other factors may influence this association. Finally, our study is cross-sectional with a specific population of Chinese children learning to read Mandarin, a language substantially different form the western languages; whether the results can be generalized to encompass an alphabetic language requires further investigation.

In conclusion, we found that the intrinsic functional connectivity between bilateral cerebellum VI and the left supramarginal gyrus was associated with RAN, and this correlation was not affected by the phonological awareness ability. These findings suggested that the relationship between the cerebellum and reading may have been related to the automatizing skill.

## Data Availability Statement

The datasets generated for this study are available on request to the corresponding author.

## Ethics Statement

The studies involving human participants were reviewed and approved by Institutional Reviews Board of the State Key Laboratory of Cognitive Neuroscience and Learning at Beijing Normal University. Written informed consent to participate in this study was provided by the participants’ legal guardian/next of kin.

## Author Contributions

GD, XM, CA, and LL carried out the study conception and design. MT, XF, MZ, and HL carried out the acquisition of data. CA and JZ carried out the analysis and interpretation of data. CA, JZ, and GD carried out the drafting of the manuscript. GD, XM, HL, and MC carried out the critical revision.

## Conflict of Interest

The authors declare that the research was conducted in the absence of any commercial or financial relationships that could be construed as a potential conflict of interest.

## Footnotes

1 http://rfmri.org/dpabi

2 http://www.diedrichsenlab.org/imaging/suit.htm

3 www.nitrc.org/projects/mricron

4 http://www.fil.ion.ucl.ac.uk/spm/

5 www.nitrc.org/projects/bnv/

## References

[B1] Alcauter S. García-Mondragón L. Gracia-Tabuenca Z. Moreno M. B. Ortiz J. J. Barrios F. A. (2017). Resting state functional connectivity of the anterior striatum and prefrontal cortex predicts reading performance in school-age children. *Brain Lang.* 174 94–102. 10.1016/j.bandl.2017.07.007 28806599

[B2] Amtmann D. Abbott R. D. Berninger V. W. (2006). Mixture growth models of RAN and RAS row by row: insight into the reading system at work over time. *Read. Writ.* 20 785–813. 10.1007/s11145-006-9041-y

[B3] Argyropoulos G. P. D. (2016). The cerebellum, internal models and prediction in ‘non-motor’ aspects of language: a critical review. *Brain Lang.* 161 4–17. 10.1016/j.bandl.2015.08.003 26320734

[B4] Baillieux H. De Smet H. J. Paquier P. F. De Deyn P. P. Marien P. (2008). Cerebellar neurocognition: insights into the bottom of the brain. *Clin. Neurol. Neurosurg.* 110 763–773. 10.1016/j.clineuro.2008.05.013 18602745

[B5] Biswal B. Zerrin Yetkin F. Haughton V. M. Hyde J. S. (1995). Functional connectivity in the motor cortex of resting human brain using echo−planar MRI. *Magn. Reson. Med.* 34 537–541. 10.1002/mrm.1910340409 8524021

[B6] Booth J. R. Wood L. Lu D. Houk J. C. Bitan T. (2007). The role of the basal ganglia and cerebellum in language processing. *Brain Res.* 1133 136–144. 10.1016/j.brainres.2006.11.074 17189619 PMC2424405

[B7] Brock J. (2013). Connectivity and cognition in autism spectrum disorders: where are the links? *Proc. Natl. Acad. Sci. U.S.A.* 110:E3973. 10.1073/pnas.1311907110 24101453 PMC3801032

[B8] Chai X. J. Berken J. A. Barbeau E. B. Soles J. Callahan M. Chen J. K. (2016). Intrinsic functional connectivity in the adult brain and success in second-language learning. *J. Neurosci.* 36 755–761. 10.1523/JNEUROSCI.2234-15.2016 26791206 PMC6602001

[B9] Chen X. Lu B. Yan C. G. (2018). Reproducibility of R−fMRI metrics on the impact of different strategies for multiple comparison correction and sample sizes. *Hum. Brain Mapp.* 39 300–318. 10.1002/hbm.23843 29024299 PMC6866539

[B10] Cummine J. Chouinard B. Szepesvari E. Georgiou G. K. (2015). An examination of the rapid automatized naming-reading relationship using functional magnetic resonance imaging. *Neuroscience* 305 49–66. 10.1016/j.neuroscience.2015.07.071 26235433

[B11] Deluca C. Golzar A. Santandrea E. Lo Gerfo E. Estocinova J. Moretto G. (2014). The cerebellum and visual perceptual learning: evidence from a motion extrapolation task. *Cortex* 58 52–71. 10.1016/j.cortex.2014.04.017 24959702

[B12] Eckert M. A. Leonard C. M. Richards T. L. Aylward E. H. Thomson J. Berninger V. W. (2003). Anatomical correlates of dyslexia: frontal and cerebellar findings. *Brain* 126 482–494. 10.1093/brain/awg026 12538414

[B13] Feng X. Li L. Zhang M. Yang X. Tian M. Xie W. (2017). Dyslexic children show atypical cerebellar activation and cerebro-cerebellar functional connectivity in orthographic and phonological processing. *Cerebellum* 16 496–507. 10.1007/s12311-016-0829-2 27785760

[B14] Fox M. D. Snyder A. Z. Vincent J. L. Corbetta M. Van Essen D. C. Raichle M. E. (2005). The human brain is intrinsically organized into dynamic, anticorrelated functional networks. *Proc. Natl. Acad. Sci. U.S.A.* 102 9673–9678. 10.1073/pnas.0504136102 15976020 PMC1157105

[B15] Hancock R. Richlan F. Hoeft F. (2017). Possible roles for fronto-striatal circuits in reading disorder. *Neurosci. Biobehav. Rev.* 72 243–260. 10.1016/j.neubiorev.2016.10.025 27826071 PMC5189679

[B16] He Q. Xue G. Chen C. Chen C. Lu Z. L. Dong Q. (2013). Decoding the neuroanatomical basis of reading ability: a multivoxel morphometric study. *J. Neurosci.* 33 12835–12843. 10.1523/JNEUROSCI.0449-13.2013 23904618 PMC3728691

[B17] Jednorog K. Marchewka A. Altarelli I. Monzalvo Lopez A. K. van Ermingen-Marbach M. Grande M. (2015). How reliable are gray matter disruptions in specific reading disability across multiple countries and languages? Insights from a large-scale voxel-based morphometry study. *Hum. Brain Mapp.* 36 1741–1754. 10.1002/hbm.22734 25598483 PMC6869714

[B18] Keren-Happuch E. Chen S.-H. A. Ho M.-H. R. Desmond J. E. (2014). A meta-analysis of cerebellar contributions to higher cognition from PET and fMRI studies. *Hum. Brain Mapp.* 35 593–615. 10.1002/hbm.22194 23125108 PMC3866223

[B19] King M. Hernandez-Castillo C. R. Poldrack R. A. Ivry R. B. Diedrichsen J. (2019). Functional boundaries in the human cerebellum revealed by a multi-domain task battery. *Nat. Neurosci.* 22 1371–1378. 10.1038/s41593-019-0436-x 31285616 PMC8312478

[B20] Koponen T. Salmi P. Eklund K. Aro T. (2013). Counting and RAN: predictors of arithmetic calculation and reading fluency. *J. Educ. Psychol.* 105 162–175. 10.1037/a0029285

[B21] Koyama M. S. Di Martino A. Zuo X. N. Kelly C. Mennes M. Jutagir D. R. (2011). Resting-state functional connectivity indexes reading competence in children and adults. *J. Neurosci.* 31 8617–8624. 10.1523/JNEUROSCI.4865-10.2011 21653865 PMC3893355

[B22] Koyama M. S. Kelly C. Shehzad Z. Penesetti D. Castellanos F. X. Milham M. P. (2010). Reading networks at rest. *Cereb. Cortex* 20 2549–2559. 10.1093/cercor/bhq005 20139150 PMC2981020

[B23] Liao C. H. Deng C. Hamilton J. Lee C. S. Wei W. Georgiou G. K. (2015). The role of rapid naming in reading development and dyslexia in Chinese. *J. Exp. Child Psychol.* 130 106–122. 10.1016/j.jecp.2014.10.002 25462035

[B24] Liao C. H. Georgiou G. K. Parrila R. (2007). Rapid naming speed and Chinese character recognition. *Read. Writ.* 21 231–253. 10.1007/s11145-007-9071-0

[B25] Lohmann G. Hoehl S. Brauer J. Danielmeier C. Bornkessel-Schlesewsky I. Bahlmann J. (2009). Setting the frame: the human brain activates a basic low-frequency network for language processing. *Cereb. Cortex* 20 1286–1292. 10.1093/cercor/bhp190 19783579

[B26] Martin A. Schurz M. Kronbichler M. Richlan F. (2015). Reading in the brain of children and adults: a meta−analysis of 40 functional magnetic resonance imaging studies. *Hum. Brain Mapp.* 36 1963–1981. 10.1002/hbm.22749 25628041 PMC4950303

[B27] McDermott K. B. Petersen S. E. Watson J. M. Ojemann J. G. (2003). A procedure for identifying regions preferentially activated by attention to semantic and phonological relations using functional magnetic resonance imaging. *Neuropsychologia* 41 293–303. 10.1016/s0028-3932(02)00162-8 12457755

[B28] Meng X. You H. Song M. Desroches A. S. Wang Z. Wei N. (2016). Neural deficits in auditory phonological processing in Chinese children with English reading impairment. *Biling. Lang. Cogn.* 19 331–346. 10.1017/s1366728915000073

[B29] Mennes M. Kelly C. Zuo X.-N. Di Martino A. Biswal B. B. Castellanos F. X. (2010). Inter-individual differences in resting-state functional connectivity predict task-induced BOLD activity. *Neuroimage* 50 1690–1701. 10.1016/j.neuroimage.2010.01.002 20079856 PMC2839004

[B30] Miro-Padilla A. Bueicheku E. Ventura-Campos N. Palomar-Garcia M. A. Avila C. (2017). Functional connectivity in resting state as a phonemic fluency ability measure. *Neuropsychologia* 97 98–103. 10.1016/j.neuropsychologia.2017.02.009 28202336

[B31] Nicolson R. I. Fawcett A. J. Dean P. (2001). Developmental dyslexia: the cerebellar deficit hypothesis. *Trends Neurosci.* 24 508–511. 10.1016/s0166-2236(00)01896-8 11506881

[B32] Noble S. Scheinost D. Constable R. T. (2019). A decade of test-retest reliability of functional connectivity: a systematic review and meta-analysis. *Neuroimage* 203:116157. 10.1016/j.neuroimage.2019.116157 31494250 PMC6907736

[B33] Norton E. S. Black J. M. Stanley L. M. Tanaka H. Gabrieli J. D. Sawyer C. (2014). Functional neuroanatomical evidence for the double-deficit hypothesis of developmental dyslexia. *Neuropsychologia* 61 235–246. 10.1016/j.neuropsychologia.2014.06.015 24953957 PMC4339699

[B34] Parrila R. Kirby J. R. McQuarrie L. (2004). Articulation rate, naming speed, verbal short-term memory, and phonological awareness: longitudinal predictors of early reading development? *Sci. Stud. Read.* 8 3–26. 10.1207/s1532799xssr0801_2

[B35] Pham A. V. Fine J. G. Semrud-Clikeman M. (2011). The influence of inattention and rapid automatized naming on reading performance. *Arch. Clin. Neuropsychol.* 26 214–224. 10.1093/arclin/acr014 21422009

[B36] Qian Y. Bi Y. Wang X. Zhang Y. W. Bi H. Y. (2016). Visual dorsal stream is associated with Chinese reading skills: a resting-state fMRI study. *Brain Lang.* 160 42–49. 10.1016/j.bandl.2016.07.007 27474853

[B37] Qian Y. Deng Y. Zhao J. Bi H. Y. (2015). Magnocellular-dorsal pathway function is associated with orthographic but not phonological skill: fMRI evidence from skilled Chinese readers. *Neuropsychologia* 71 84–90. 10.1016/j.neuropsychologia.2015.03.024 25813780

[B38] Raberger T. Wimmer H. (2003). On the automaticity/cerebellar deficit hypothesis of dyslexia: balancing and continuous rapid naming in dyslexic and ADHD children. *Neuropsychologia* 41 1493–1497. 10.1016/s0028-3932(03)00078-2 12849767

[B39] Raschle N. M. Zuk J. Gaab N. (2012). Functional characteristics of developmental dyslexia in left-hemispheric posterior brain regions predate reading onset. *Proc. Natl. Acad. Sci. U.S.A.* 109 2156–2161. 10.1073/pnas.1107721109 22308323 PMC3277560

[B40] Raven J. C. (1998). *Raven’s Progressive Matrices and Vocabulary Scales.* Oxford: Oxford Pyschologists Press.

[B41] Shah L. M. Cramer J. A. Ferguson M. A. Birn R. M. Anderson J. S. (2016). Reliability and reproducibility of individual differences in functional connectivity acquired during task and resting state. *Brain Behav.* 6:e00456. 10.1002/brb3.456 27069771 PMC4814225

[B42] Shu H. McBride-Chang C. Wu S. Liu H. (2006). Understanding Chinese developmental dyslexia: morphological awareness as a core cognitive construct. *J. Educ. Psychol.* 98 122–133. 10.1037/0022-0663.98.1.122

[B43] Shu H. Peng H. McBride−Chang C. (2008). Phonological awareness in young Chinese children. *Dev. Sci.* 11 171–181. 10.1111/j.1467-7687.2007.00654.x 18171377

[B44] Sokolov A. A. Miall R. C. Ivry R. B. (2017). The Cerebellum: adaptive prediction for movement and cognition. *Trends Cogn. Sci.* 21 313–332. 10.1016/j.tics.2017.02.005 28385461 PMC5477675

[B45] Song S. Georgiou G. K. Su M. Hua S. (2015). How well do phonological awareness and rapid automatized naming correlate with Chinese reading accuracy and fluency? A meta-analysis. *Sci. Stud. Read.* 20 99–123. 10.1080/10888438.2015.1088543

[B46] Stoodley C. J. Schmahmann J. D. (2009). Functional topography in the human cerebellum: a meta-analysis of neuroimaging studies. *Neuroimage* 44 489–501. 10.1016/j.neuroimage.2008.08.039 18835452

[B47] Stoodley C. J. Stein J. F. (2011). The cerebellum and dyslexia. *Cortex* 47 101–116. 10.1016/j.cortex.2009.10.005 20060110

[B48] Stoodley C. J. Stein J. F. (2013). Cerebellar function in developmental dyslexia. *Cerebellum* 12 267–276. 10.1007/s12311-012-0407-1 22851215

[B49] Turkeltaub P. E. Gareau L. Flowers D. L. Zeffiro T. A. Eden G. F. (2003). Development of neural mechanisms for reading. *Nat. Neurosci.* 6 767–773. 12754516 10.1038/nn1065

[B50] Tzourio-Mazoyer N. Landeau B. Papathanassiou D. Crivello F. Etard O. Delcroix N. (2002). Automated anatomical labeling of activations in SPM using a macroscopic anatomical parcellation of the MNI MRI single-subject brain. *Neuroimage* 15 273–289. 10.1006/nimg.2001.0978 11771995

[B51] Wang L. LaViolette P. O’Keefe K. Putcha D. Bakkour A. Van Dijk K. R. (2010). Intrinsic connectivity between the hippocampus and posteromedial cortex predicts memory performance in cognitively intact older individuals. *Neuroimage* 51 910–917. 10.1016/j.neuroimage.2010.02.046 20188183 PMC2856812

[B52] Wang X. Han Z. He Y. Liu L. Bi Y. (2012). Resting-state functional connectivity patterns predict Chinese word reading competency. *PLoS One* 7:e44848. 10.1371/journal.pone.0044848 23028644 PMC3454382

[B53] Welsh J. A. Nix R. L. Blair C. Bierman K. L. Nelson K. E. (2010). The development of cognitive skills and gains in academic school readiness for children from low-income families. *J. Educ. Psychol.* 102 43–53. 10.1037/a0016738 20411025 PMC2856933

[B54] Wolf M. Bowers P. G. (1999). The double-deficit hypothesis for the developmental dyslexias. *J. Educ. Psychol.* 91 415–438. 10.1037/0022-0663.91.3.415

[B55] Xia M. Wang J. He Y. (2013). BrainNet viewer: a network visualization tool for human brain connectomics. *PLoS One* 8:e68910. 10.1371/journal.pone.0068910 23861951 PMC3701683

[B56] Xu J. Rees G. Yin X. Song C. Han Y. Ge H. (2014). Spontaneous neuronal activity predicts intersubject variations in executive control of attention. *Neuroscience* 263 181–192. 10.1016/j.neuroscience.2014.01.020 24447598

[B57] Yan C. G. Wang X. D. Zuo X. N. Zang Y. F. (2016). DPABI: data processing & analysis for (resting-state) brain imaging. *Neuroinformatics* 14 339–351. 10.1007/s12021-016-9299-4 27075850

[B58] Yang H. Yang S. Kang C. (2014). The relationship between phonological awareness and executive attention in Chinese-English bilingual children. *Cogn. Dev.* 30 65–80. 10.1016/j.cogdev.2013.11.003

[B59] Yeung P.-S. Ho C. S.-H. Chik P. P.-M. Lo L.-Y. Luan H. Chan D. W.-O. (2011). Reading and spelling Chinese among beginning readers: what skills make a difference? *Sci. Stud. Read.* 15 285–313. 10.1080/10888438.2010.482149

[B60] Zacks J. M. Ferstl E. C. (2016). “Discourse comprehension,” in *Neurobiology of Language*, eds Hickok G. Small S. L. (Amsterdam: Elsevier), 661–673.

[B61] Zang Y. F. He Y. Zhu C. Z. Cao Q. J. Sui M. Q. Liang M. (2007). Altered baseline brain activity in children with ADHD revealed by resting-state functional MRI. *Brain Dev.* 29 83–91. 10.1016/j.braindev.2006.07.002 16919409

[B62] Zhang M. Li J. Chen C. Xue G. Lu Z. Mei L. (2014). Resting-state functional connectivity and reading abilities in first and second languages. *Neuroimage* 84 546–553. 10.1016/j.neuroimage.2013.09.006 24055555 PMC3849213

[B63] Ziegler J. C. Bertrand D. Tóth D. Csépe V. Reis A. Faísca L. (2010). Orthographic depth and its impact on universal predictors of reading. *Psychol. Sci.* 21 551–559. 10.1177/0956797610363406 20424101

