# Supplementary material for: Intrinsic Cerebro-Cerebellar Functional Connectivity Reveals the Function of Cerebellum VI in Reading-Related Skills
Source: Front Psychol. 2020 Mar 20;11:420. doi: 10.3389/fpsyg.2020.00420 (PMC7099000; doi:10.3389/fpsyg.2020.00420)
Supplement: Supplementary file 1 [file Data_Sheet_1.docx]

Supplementary Material

**Supplementary Table 1**. The correlation between the cerebro-cerebellar resting-state functional connectivity and PA/RAN when with Raven’s IQ as the covariate.

| Seed regions | Reading-related tests | Cerebral cortex | Lateral | MNI coordinates | | | T value | Voxels |
| --- | --- | --- | --- | --- | --- | --- | --- | --- |
|  |  |  |  | x | y | Z |  |  |
| Left cerebellum VI | RAN | Postcentral gyrus | Left | -60 | -15 | 24 | 5.00 | 73 |
| Left cerebellum VI | PA | / |  |  |  |  |  |  |
| Right cerebellum VI | PA | *Insular | Left | -51 | -9 | 12 | 4.61 | 53 |
| Right cerebellum VI | RAN | *Supramarginal gyrus | Left | -57 | -18 | 24 | 5.32 | 43 |

Notes. T-value, and MNI coordinate are for the peak voxel in each cluster only. Results were reported at a threshold of an individual voxel-level *p* < 0.001, cluster-level *p* < 0.05, corrected by GRF. *a loose threshold of an individual voxel *p* < 0.001 uncorrected. RAN = rapid automatized naming, PA = phonological awareness.

**Supplementary Table 2**. The correlation between the cerebro-cerebellar resting-state functional connectivity and PA/RAN when with one of them as the dependent variable and the other as the covariate.

| Seed regions | Reading-related tests | Cerebral cortex | Lateral | MNI coordinates | | | T value | Voxels |
| --- | --- | --- | --- | --- | --- | --- | --- | --- |
|  |  |  |  | x | y | Z |  |  |
| Left cerebellum VI | RAN | Supramarginal gyrus | Left | -63 | -21 | 27 | 4.42 | 25 |
| Left cerebellum VI | PA | / |  |  |  |  |  |  |
| Right cerebellum VI | PA | / |  |  |  |  |  |  |
| Right cerebellum VI | RAN | Supramarginal gyrus | Left | -60 | -21 | 24 | 4.30 | 16 |

Notes. T-value, and MNI coordinate are for the peak voxel in each cluster only. Results were reported with the threshold of an individual voxel *p* < 0.001 uncorrected. RAN = rapid automatized naming, PA = phonological awareness.

**Supplementary Table 3**. The correlation between the cerebro-cerebellar resting-state functional connectivity and PA/RAN when with IQ, PA, and RAN as the covariates.

| Seed regions | Reading-related tests | Cerebral cortex | Lateral | MNI coordinates | | | T value | Voxels |
| --- | --- | --- | --- | --- | --- | --- | --- | --- |
|  |  |  |  | x | y | Z |  |  |
| Left cerebellum VI | RAN | Supramarginal gyrus | Left | -63 | -21 | 27 | 4.34 | 21 |
| Left cerebellum VI | PA | / |  |  |  |  |  |  |
| Right cerebellum VI | PA | / |  |  |  |  |  |  |
| Right cerebellum VI | RAN | Supramarginal gyrus | Left | -60 | -21 | 24 | 4.23 | 14 |

Notes. T-value, and MNI coordinate are for the peak voxel in each cluster only. Results were reported with the threshold of an individual voxel *p* < 0.001 uncorrected. RAN = rapid automatized naming, PA = phonological awareness.

**
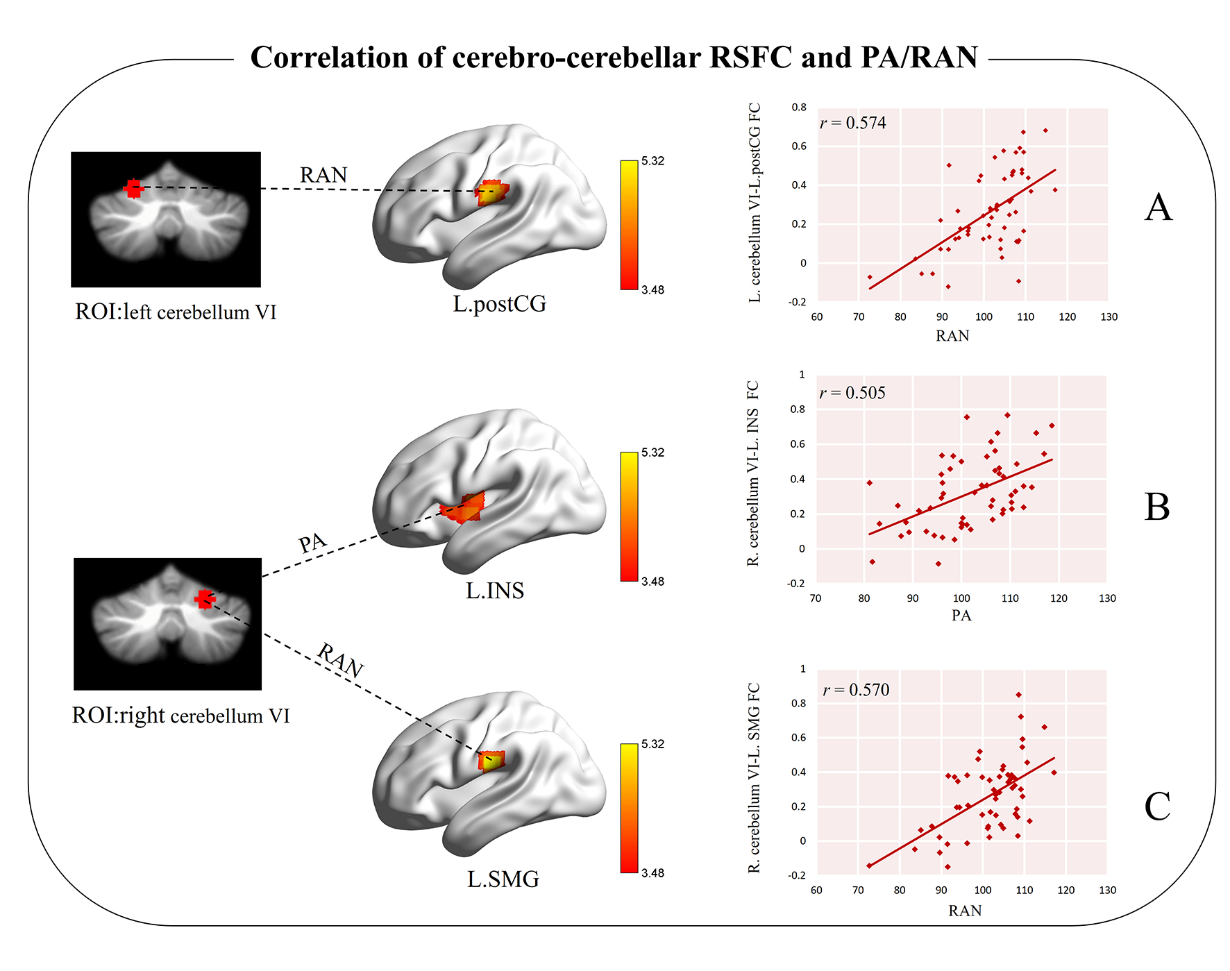
**

**Supplementary Figure 1.** The correlation between cerebro-cerebellar functional connectivity and PA/RAN when with Raven’s IQ as the covariate. (**A**) Functional connectivity between left cerebellum VI and left postCG (extend to the left SMG) was positively correlated with RAN. (**B**) Functional connectivity between right cerebellum VI and left INS was positively correlated with PA. (**C**) Functional connectivity between right cerebellum VI and left SMG was positively correlated with RAN under a loose threshold. L.postCG = the left postcentral gyrus, L.SMG = the left supramarginal gyrus, L.INS = the left insula, RAN = rapid automatized naming, PA = phonological awareness.

**
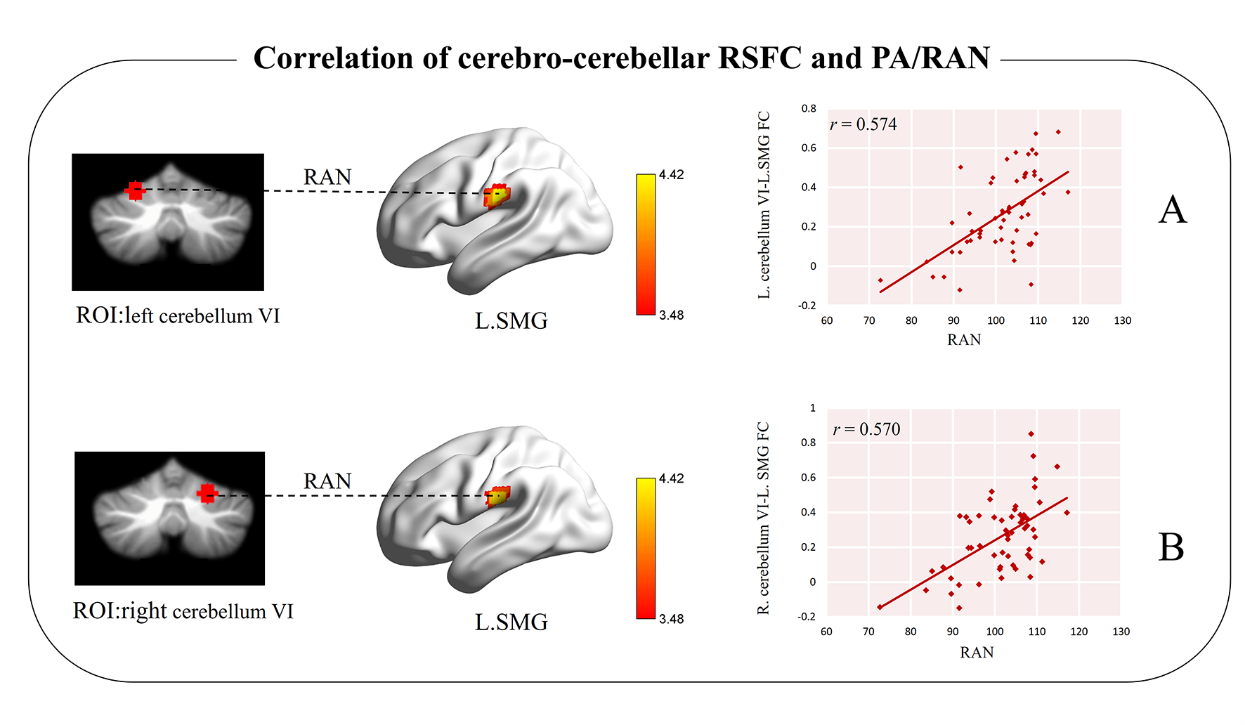
**

**Supplementary Figure 2.** The correlation between the cerebro-cerebellar resting-state functional connectivity and PA/RAN when with one of them as the dependent variable and the other as the covariate. (**A**) Functional connectivity between left cerebellum VI and left SMG was positively correlated with RAN under a loose threshold. (**B**) Functional connectivity between right cerebellum VI and left SMG was positively correlated with RAN under a loose threshold. L.SMG = the left supramarginal gyrus, L.INS = the left insula, RAN = rapid automatized naming, PA = phonological awareness.

**
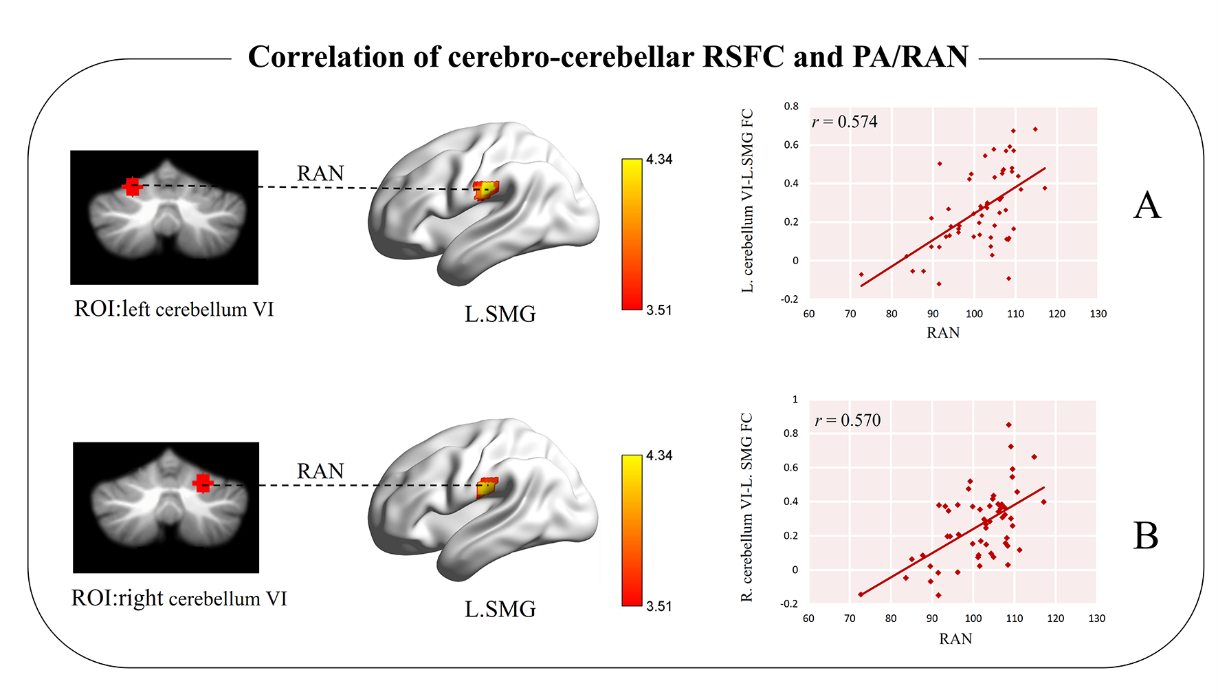
**

**Supplementary Figure 3.** The correlation between the cerebro-cerebellar resting-state functional connectivity and PA/RAN when with IQ, PA, and RAN as the covariates. (**A**) Functional connectivity between left cerebellum VI and left SMG was positively correlated with RAN under a loose threshold. (**B**) Functional connectivity between right cerebellum VI and left SMG was positively correlated with RAN under a loose threshold. L.SMG = the left supramarginal gyrus, L.INS = the left insula, RAN = rapid automatized naming, PA = phonological awareness.

**
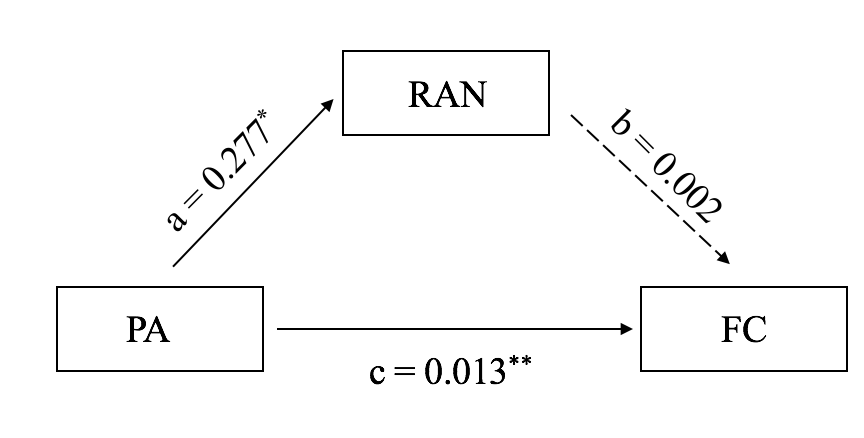
**

**Supplementary Figure 4.** The additional mediation analysis indicated that that although phonological awareness (PA) predicts functional connectivity between the right cerebellum VI and the left insular (c-path), PA predicts rapid automatized naming (RAN) (a-path), but RAN to FC (b-path) in this mediation model is not significant, suggesting that the RSFC of cerebellum VI could not be affected by PA via RAN. Mediation results are shown as regression coefficients. **p* < 0.05; ****p* < 0.001.
